# Real-Time Forecast of Influenza Outbreak Using Dynamic Network Marker Based on Minimum Spanning Tree

**DOI:** 10.1155/2020/7351398

**Published:** 2020-10-01

**Authors:** Kun Yang, Jialiu Xie, Rong Xie, Yucong Pan, Rui Liu, Pei Chen

**Affiliations:** ^1^School of Computer Science and Engineering, South China University of Technology, Guangzhou 510006, China; ^2^Department of Biostatistics, University of North Carolina at Chapel Hill, 27514, USA; ^3^School of Information, Guangdong University of Finance and Economics, Guangzhou 510320, China; ^4^Guangdong Science and Technology Infrastructure Center, Guangzhou 510033, China; ^5^School of Mathematics, South China University of Technology, Guangzhou 510640, China

## Abstract

The influenza pandemic is a wide-ranging threat to people's health and property all over the world. Developing effective strategies for predicting the influenza outbreak which may prevent or at least get ready for a new influenza pandemic is now a top global public health priority. Owing to the complexity of influenza outbreaks that are usually involved with spatial and temporal characteristics of both biological and social systems, however, it is a challenging task to achieve the real-time monitoring of influenza outbreaks. In this study, by exploring the rich dynamical information of the city network during influenza outbreaks, we developed a computational method, the minimum-spanning-tree-based dynamical network marker (MST-DNM), to identify the tipping point or critical stage prior to the influenza outbreak. With historical records of influenza outpatients between 2009 and 2018, the MST-DNM strategy has been validated by accurate predictions of the influenza outbreaks in three Japanese cities/regions, respectively, i.e., Tokyo, Osaka, and Hokkaido. These successful applications show that the early-warning signal was detected 4 weeks on average ahead of each influenza outbreak. The results show that our method is of considerable potential in the practice of public health surveillance.

## 1. Introduction

Influenza, a seasonal, contagious, and widespread respiratory illness, has always been a huge threat to people's health. According to the World Health Organization, up to 650,000 deaths annually are associated with respiratory diseases caused by seasonal influenza. In the United States, the influenza pandemic leads to an average of 610,660 deaths per year and 3.1 million hospitalized days [1]. It is estimated that the total economic burden caused by influenza reaches 81.7 billion US dollars each year [2]. Therefore, from both public health and economic perspective, it is crucial to detect the early-warning signal of imminent influenza outbreak so that timely preventive measures can be carried out to prevent a new influenza pandemic or at least reduce the magnitude of influenza outbreaks [3, 4]. However, it is usually a challenging task to predict the influenza outbreak due to the complexity of its temporal and spatial characteristics. First, the records of worldwide influenza pandemics showed that each outbreak differed from the others with respect to etiologic agents, epidemiology, and disease severity [5]. Second, there is a major obstacle for most developing countries to deploy influenza forecasts, that is, the national surveillance system for infectious disease could be either too costly or inaccurate [6]. Therefore, it is of great concern to develop a cost-effective computational method for predicting the outbreak of influenza only based on the available data.

In this study, by exploring the rich dynamical information provided by high-dimensional records of clinic hospitalization data, we developed a practical computational method, i.e., the minimum-spanning-tree-based dynamical network marker (MST-DNM), to quantitatively measure the dynamical change of a city network and thus detect the early-warning signal of an influenza outbreak. The theoretical basis of MST-DNM is our recently proposed concept, the so-called dynamical network marker (DNM) [7], which is a dominant group of variables satisfying three generic properties for the impending critical transitions, that is, (1) the correlation between any pair of members in the DNM group rapidly increases; (2) the correlation between one member of the DNM group and any other non-DNM member rapidly decreases; (3) the standard deviation or coefficient of variation for any member in the DNM group drastically increases. Different from traditional biomarkers, the DNM method is aimed at detecting the early-warning signal of the critical state before the occurrence of a catastrophic event, by mining the critical information from high-dimensional time series data [7, 8]. The DNM method has been applied to real-world datasets and successfully identified the critical states for a number of biological processes, such as the critical state of cell differentiation [9], the tipping point during the cell fate decision process [10], the critical transition in the immune checkpoint blockade-responsive tumor [11], the multistage deteriorations of T2D [12], acute lung injury [13], HCV-induced liver cancer [14], cancer metastasis [15], and other complex diseases [15–19]. However, to accurately predict the influenza outbreak, a new computational method is required to explore and measure the criticality from a network perspective by considering the geographic information of a city.

**Figure 1:**
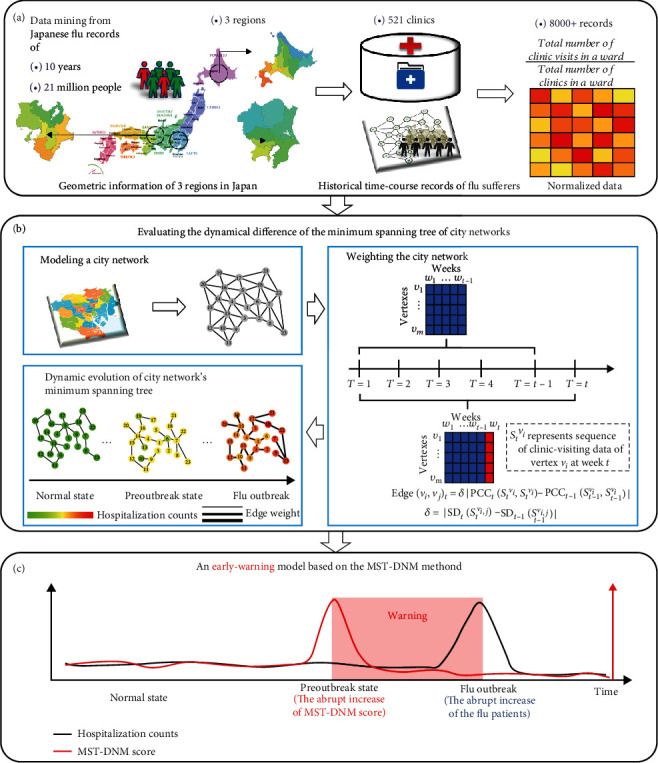
Schematic illustration of detecting the early-warning signal of influenza outbreak based on MST-DNM. (a) The historical records of clinic visits caused by influenza between 1 January 2009 and 1 May 2019 were collected from three regions of Japan, including Tokyo, Osaka, and Hokkaido. (b) Through building a city network, weighting, and the changes of the minimum spanning tree of this network, the MST-DNM method can monitor in real time the progress of the influenza and issue early-warning signals in a timely manner. (c) Based on the MST-DNM method, the outbreak process of influenza could be divided into three states, i.e., the normal state, the preoutbreak state, and the flu outbreak state. The abrupt increase of MST-DNM score means the arrival of the preoutbreak state.

The MST-DNM is a novel network-based computational method combined with minimum spanning tree for accurate detection of early-warning signal to the influenza outbreak. The spread of infectious diseases in a region is described as the dynamical evolution of a nonlinear system, while the influenza outbreak is regarded as a qualitative state transition of the dynamical system. Without loss of generality, there are three states for the influenza outbreak (Figure 1), that is, a normal state with high stability and robustness to disturbances, standing for the period with few clinic visits; a preoutbreak state (critical state) with low resilience and high convertibility, representing the critical stage just before the emergence of massive clinic visits; and an outbreak state with high stability and robustness, which is an irreversible state or severe flu pandemic with massive clinic visits. Clearly, identifying the preoutbreak state is crucial in influenza control since timely management may greatly reduce the magnitude and duration of the influenza outbreak. Specifically, by combining the geographically adjacent information, transportation, population, and the number of clinics of each city district, we constructed a city network with edge weights which were assigned as the correlation between the clinic visit numbers of two adjacent districts. By analyzing the dynamical transmission of influenza in the city network, the proposed MST-DNM can accurately identify the preoutbreak state and thus early signal influenza outbreaks or potential pandemics. Specifically, the MST-DNM method was employed to probe useful dynamical information in a city network, which is modeled based on geographic location and traffic conditions, from the high-dimensional clinic-visiting data of influenza, which are from 175 clinics distributed in 23 wards of Tokyo, Japan, 139 clinics distributed in 30 cities of Hokkaido, Japan, and 197 clinics distributed in 11 wards of Osaka, Japan. Clearly, such real-time data could be much more readily available for a large-scale surveillance system. The results indicate that the MST-DNM method is capable of monitoring the infection process of the flu in real time and timely identifying the warning signal before the outbreak of influenza. Moreover, by analyzing the dynamic changes of the minimum spanning tree in a city network, it provides a new approach to study the epidemic spread in a city. Therefore, this method is of great applicable potential in setting up a real-time surveillance system, which could be greatly favorable for preventive care or the implementation of interventions to a health epidemic.

## 2. Materials and Methods

### 2.1. Theoretical Background

The influenza spread and outbreak is a complex dynamic process of a nonlinear system. According to the DNM theory, when a complex system approaches to a tipping point or critical transition point, there is a dominant group, i.e., the DNM, which satisfies the following three essential properties [7]:

- The correlation (PCC_in_) between each pair of members in the DNM group dramatically increases
- The correlation (PCC_out_) between a member of the DNM group and a non-DNM member rapidly decreases
- The standard deviation (SD_in_) for each member in the DNM group drastically increases

In general, the above properties can be roughly understood as that the emergence of the DNM group with violent fluctuation and high correlation signifies the upcoming critical transition. Thus, these properties can be utilized as three criteria to identify the critical state of a complex biological system.

Based on the DNM theory, we developed the MST-DNM method in order to accurately predict the early-warning signal to the influenza outbreak, by combining with the minimum spanning tree in a city network. According to our method, the evolution process of flu outbreak could be modeled as three diverse stages or states (Figure 1): (i) the normal stage, which is a stable state with high resilience; (ii) the preoutbreak stage, which is an unstable critical state with low resilience; this critical state is the limit of the normal state and at the edge of transition into an epidemic outbreak of influenza; and (iii) the outbreak stage, which is a steady and irreversible stage with a large number of clinic visits caused by influenza. It would bring heavy economic burdens to people and society and strongly impact the existing social health security system once in this status. Consequently, it is crucial to identify the warning signal of the preoutbreak state to prevent people and society from the catastrophic flu outbreak in some effective measures.

### 2.2. Algorithm

**Figure 2:**
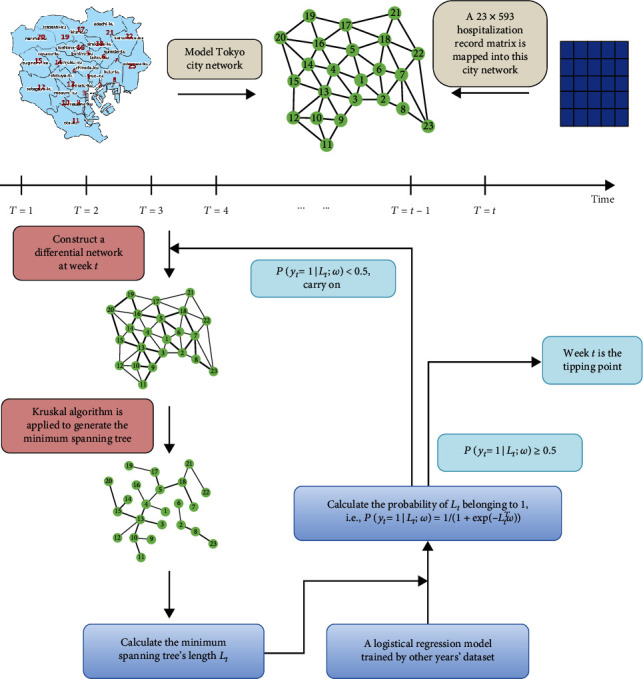
The overall algorithm structure of MST-DNM method. First, model a city network based on its administrative divisions and the geographical relationship and map the corresponding clinic-visiting record matrix into the city network. Then, regard a week *t* as a candidate tipping point, weight the city network, and calculate its minimum spanning tree's length as the MST-DNM score *L*_*t*_. Finally, according to a logistic regression model trained by other years' dataset, calculate the probability of *L*_*t*_ belonging to 1, i.e., *P*(*y*_*t*_ = 1│*L*_*t*_; *ω*) = 1/(1 + exp(−*L*_*t*_^*T*^ *ω*)). If this probability is greater than or equal to 0.5, week *t* is considered as the tipping point. Otherwise, week *t* is classified as the normal state, and the algorithm carries on with week *t* + 1.

The sketch of the MST-DNM method is presented in Figure 2. First, it is noted that the MST-DNM method is applied to a city network for monitoring the influenza spread and outbreak in such a city. Therefore, the first step of our method is to model a city network by combining the information of geographically adjacent relationship, transportation, population, and the number of clinics of each city district. Then, a weight was assigned to each edge of the city network, which was the correlation between the numbers of clinic visits of two adjacent districts. Based on such weighted city network, our method is implemented. Specifically, in order to detect the critical state of influenza outbreak, the procedure of the MST-DNM method can be described as the following detailed steps. Its pseudocode is illustrated in Algorithm 1.

#### 2.2.1. Modeling a City Network Structure

A city network is modeled based on its administrative divisions' geographic location and their adjacent information. As demonstrated in Figure 2, for example, there are 23 districts in Tokyo, so that 23 nodes are added into the Tokyo city network. Furthermore, the edges between nodes in the network are established based on the adjacency relations of those corresponding districts.

#### 2.2.2. Data Preprocessing

For each district of a city, it is necessary that the raw data which is weekly based should be averaged in terms of the total number of clinics within the district, owing to the enormous discrepancy of the number of visits between different clinics. Afterwards, the processed data is mapped to the city network.

#### 2.2.3. Implement

The city network can be represented as a graph *G* = (*V*, *E*), where *V* = {*v*_*i*_}_*i*=1_^*M*^ is a set of *M* vertexes in this network and *E* = {*e*_*k*_}_*k*=1_^*N*^ is a set of *N* edges in this network. There are the following procedures.

First, we consider the number of clinic visits per week of a district as a sample *s*, forming a series of time series data. In other words, when the city network is at week *t*, there is a sequence of clinic-visiting data *S*_*t*_^*v*_*i*_^ = {*s*_1_,*s*_2_,…, *s*_*t*_} for each vertex *v*_*i*_.

Second, for each edge *e*_*k*_ of the city network at week *t*, calculate the correlations between the two vertexes *v*_*i*_, *v*_*j*_ of this edge to give it a weight *W*_*t*_^*k*^:

(1) $$\begin{array}{c} W_{t}^{k}=\delta \left\|{\text{PCC}}_{t}\left(S_{t}^{v_{i}},S_{t}^{v_{j}}\right)\;|\;-\;|\;{\text{PCC}}_{t-1}\left(S_{t-1}^{v_{i}},S_{t-1}^{v_{j}}\right)\right\|, \end{array}$$

where PCC_*t*_(*S*_*t*_^*v*_*i*_^, *S*_*t*_^*v*_*j*_^) represents the Pearson correlation coefficient (PCC) between the two vertexes *v*_*i*_, *v*_*j*_ at week *t* and PCC_*t*−1_(*S*_*t*−1_^*v*_*i*_^, *S*_*t*−1_^*v*_*j*_^) represents the Pearson correlation coefficient between the two vertexes *v*_*i*_, *v*_*j*_ at week *t* − 1, and parameter *δ* is of the following form:

(2) $$\begin{array}{c} \delta =\left\|\;|\;{\text{SD}}_{t}\left(S_{t}^{v_{i},v_{j}}\right)\;|\;-\;|\;{\text{SD}}_{t-1}\left(S_{t-1}^{v_{i},v_{j}}\right)\right\|, \end{array}$$

where SD_*t*_(*S*_*t*_^*v*_*i*_,*v*_*j*_^) represents the standard deviation (SD) of all simple data of the two vertexes of this edge *e*_*k*_ at week *t* and SD_*t*−1_(*S*_*t*−1_^*v*_*i*_,*v*_*j*_^) represents the standard deviation of all simple data of the two vertexes of this edge *e*_*k*_ at week *t* − 1. After this step, we have obtained a set of weighted differential network {*N*_1_,*N*_2_,…, *N*_*t*_, ⋯}.

Third, when the city network is at week *t*, in order to better describe its evolution as the number of visits changes, it is required to obtain its minimum spanning tree. In this study, Kruskal's algorithm is applied to the time-specific weighted differential network *N*_*t*_ (such network is generated specifically for a time point) to obtain its minimum spanning tree MST_*t*_. The detailed flow of Kruskal's algorithm is presented in Algorithm 2. Then, we can calculate the weight sum *L*_*t*_ of this minimum spanning tree as the MST-DNM score:

(3) $$\begin{array}{c} L_{t}=\overset{K}{\underset{i=1}{\sum }}{\text{Weight}}_{i}, \end{array}$$

where Weight_*i*_ represents the weight of edge *e*_*i*_ in MST_*t*_ and *K* represents the total number of edges of MST_*t*_.

In the ideal case, when the network system approaches a tipping point, there are the following two properties for the relationship between nodes in the network:

- The nodes in the city network are all DNM members. The standard deviation of these members and the Pearson's correlation coefficient between these members both dramatically increase
- There are DNM and non-DNM members in the city network. The standard deviation of the DNM members dramatically increases, but the Pearson's correlation coefficient between DNM members and non-DNM members decreases significantly, i.e., its absolute value increases significantly

Meanwhile, the proposed city network's MST-DNM score *L*_*t*_ is based on the standard deviations of these DNM members and their Pearson's correlation coefficients; thus, it could be employed as an index for quantitatively analyzing the significant change of the city network, thus detecting the warning signal of the critical point.

#### 2.2.4. Identifying the Critical State

After the above procedure, it is possible to quantitatively analyze and monitor the dynamical process of influenza spreading based on the indicator *L*_*t*_. Nevertheless, it is still a tough task to confirm the tipping point. In some previous studies, the fold-change thresholds were used to detect the warning signal [20, 21]. However, such empirical or tunable threshold is not a universal method for different data or network structures. In this study, the logistic regression is applied to determine the appearance of the tipping point, which is widely employed in the biological field [22] due to its intrinsic advantage that the threshold is determined by the data itself. In view of the sufficient training data (several years of clinic-visiting records), the learning-based approach would be an optimal option.

Logistic regression, which essentially is a linear regression model based on the sigmoid function, is used to analyze the dataset with duality to explore relationship between its internal independent variables, i.e., solving two-class (0 or 1) problems. Assume a dataset with *m* samples and *n* feature and each sample with a binary label. Then, we will get a sample matrix *X* = (*x*_1_, *x*_2_, ⋯,*x*_*m*_)^*T*^ ∈ *R*^*mn*^, where *x*_*i*_ is a column matrix with *n* features, and corresponding label *Y* = (*y*_1_, *y*_2_, ⋯, *y*_*m*_), where *y*_*i*_ represents a binary label (0 or 1). Usually, we will add an extra item to *X* as a bias; therefore, each *x*_*i*_ is represented by *x*_*i*_ = (*x*_*i*_^0^, *x*_*i*_^1^, ⋯, *x*_*i*_^*n*^). Then, the sigmoid function is applied to calculate the probability for *x*_*i*_ belonging to 1:

(4) $$\begin{array}{c} P\left(y_{i}=1\;|\;x_{i};\omega \right)=\frac{1}{1+\exp\left(-x_{i}^{T}\omega \right)}. \end{array}$$

According to the above form, the key to the logistic regression model is to train a suitable parameter *ω* based on the given sample *X* and label *Y*. Therefore, the following loss function based on the negative log-likelihood is applied to optimize our logistic regression model to obtain suitable *ω*:

(5) $$\begin{array}{c} L\left(\omega \right)=-\overset{n}{\underset{i=1}{\sum }}\left(y_{i}\log x_{i}^{T}\omega +\left(1-y_{i}\right)\log\left(1-x_{i}^{T}\omega \right)\right)+{\left|\left|\omega \right|\right|}_{1}. \end{array}$$

In order to prevent our model from overfitting, the  *l*_1_ norm was added into the loss function. Since there is no direct solution to this loss function at present, we used coordinate descent to minimize this loss function with respect to *ω*.

In this study, we used the MST score of each week as *X* and the relevant state as label *Y*, where 1 represents the critical state and 0 represents others. For a certain year, the logistic regression model is trained by other years' datasets; we tested whether the week *T* = *t* is the tipping point. As long as the probability of *x*_*t*_ belonging to 1, i.e., *P*(*y*_*t*_ = 1 | *x*_*t*_; *ω*) = 1/(1 + exp(−*x*_*t*_^*T*^*ω*)), is greater than 0.5, this week is considered to be the critical state. Otherwise, this week is classified as the normal state. Then, the week *T* = *t* + 1 is selected as the new test point to carry on.

## 3. Results and Discussion

### 3.1. Predict the Outbreak of Seasonal Influenza in Tokyo

It is usually too complicated to mathematically express the influenza transmission kinetics before a sudden outbreak, because the influenza spread involves massive parameters from both biological and social systems. Based on the dynamical systems theory, there exists a so-called bifurcation point when there are dramatic fluctuations or a qualitative transformation in a network from its normal status [19, 23]. It means that the state transition of a dynamical system would gradually be restricted in a one- or two-dimensional space so that the system can be simply expressed and understood while approaching the bifurcation point [7]. According to this theory, it is achievable to develop a general method to detect the tipping point of influenza outbreak only based on the observed data.

As shown in Figure 1, we collected the historical clinic-visiting data caused by influenza from clinics in 23 districts of Tokyo, Japan, from January 1, 2009, to May 31, 2019. It can be regarded as the outbreak point of flu when the number of total clinic visits reaches the peak in each year. According to the proposed method, MST-DNM, the following procedures will be carried out to identify the critical state of flu outbreak in Tokyo. First, we modeled a 23-node network according to the geographic location of 23 wards and their adjacency. Second, we mapped the clinic-visiting numbers into corresponding nodes, assign weights (i.e., the correlations between two adjacent nodes, the detailed calculation is in Materials and Methods) to edges, and calculate the weight sum of the minimum spanning tree of this network for each week. Finally, an analyzed data matrix constituted by MST-DNM scores was obtained, which was employed to train a logistic regression through leave-one-out cross-validation and further detect the tipping point of influenza for each year.

**Figure 3:**
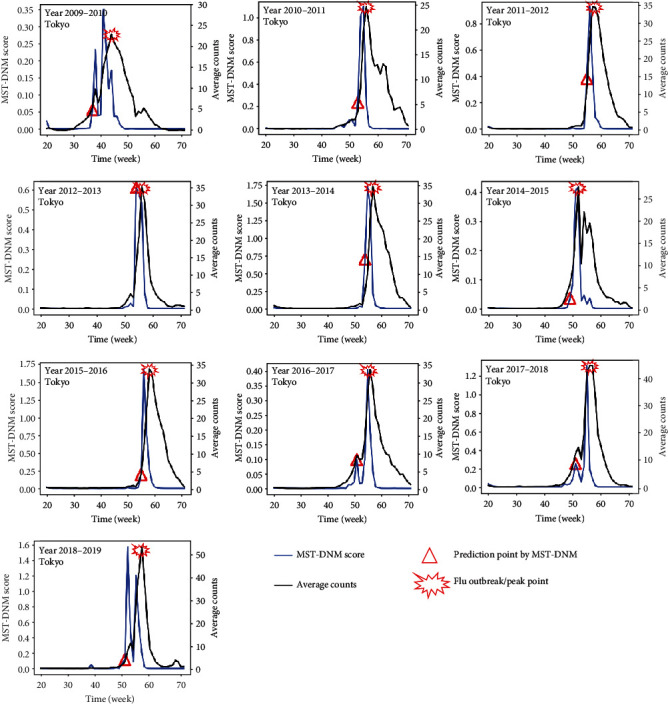
The predictions of annual influenza outbreak in Tokyo city between 2009 and 2019. For each year, our MST-DNM method timely issues the early-warning signal of influenza outbreak only based on the clinic-visiting information. For each figure, the *x*-axis represents the time evolution from the 20th week to the 72nd week (roughly a seasonal-outbreak period), and the *y*-axis represents the MST-DNM score and average number of clinic visits, respectively. The red hollow triangle represents the early-warning signal detected by the MST-DNM method, and the explosion symbol is the actual outbreak point of influenza, i.e., the peak of the clinic-visiting number.

As presented in Figure 3, the early-warning signals of the seasonal influenza outbreak were detected by our MST-DNM method. It can be seen that the flu outbreak of each year is quite regular except year 2009. The worldwide large-scale outbreak of influenza A (H1N1) in 2009, which was reported first in Mexico, led to a massive long-term outbreak of influenza in Tokyo. It is explicit that the peak of *L*_*t*_ appears earlier than the peak of the clinic-visiting counts for 4 weeks on average. Therefore, before the outbreak of influenza, our MST-DNM score is quite sensitive and the index *L*_*t*_ increases drastically, which implies the appearance of critical state of the influenza outbreak.

**Figure 4:**
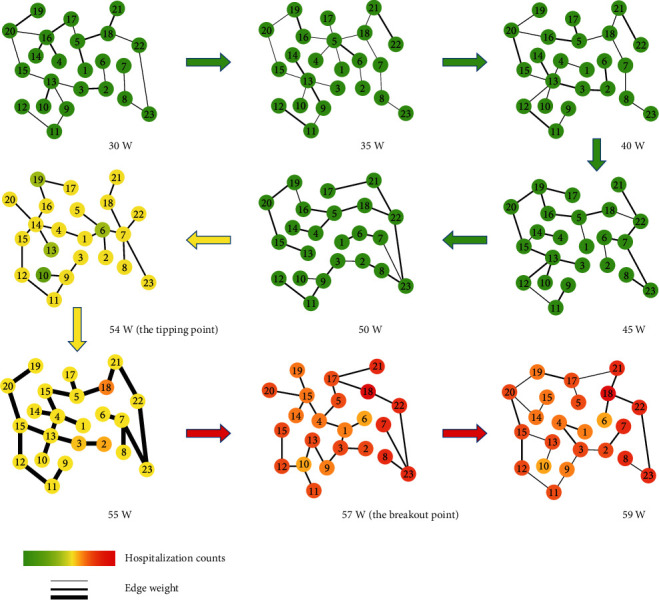
The dynamic evolution of the minimum spanning tree of the city network in Tokyo during years 2013-2014. The nodes are colored by the average number of clinic visits of the corresponding district, and the thickness of the edges represents the correlations between corresponding nodes (the detailed calculation is in Materials and Methods). It is clear that the edges become thicker before the nodes turn red in week 54, which indicates that the early-warning signals from our method appear before the flu outbreak.

In order to better demonstrate the dynamical process of the influenza spread in the network level, the evolutions of minimum spanning tree of the city network can also be presented. As shown in Figure 4, it is seen that there are almost no influenza cases at each node/ward and the correlations between these adjacent nodes/wards are relatively low at the beginning. In the city network, when the correlations between the adjacent nodes/wards drastically increase, which are the necessary conditions of the DNM features, it indicates that the influenza spread in this city is closed to its outbreak point. Furthermore, the edges of the minimum spanning tree become thicker before the nodes turn red in week 54, which means that the early-warning signals of our method appear before the flu outbreak point. The dynamical evolution of minimum spanning tree of the city network illustrates that the system based on the MST-DNM method is able to monitor the whole process of influenza outbreak in real time and issue an early-warning signal in time.

### 3.2. Application of MST-DNM in Osaka and Hokkaido

In order to illustrate the universality of our MST-DNM method, we also applied it to detect the early-warning signals of flu outbreak in Hokkaido and Osaka. Similar to the processing flow in Tokyo city, a 30-node city network was modeled for Hokkaido region and an 11-node city network for Osaka city. Then, we mapped the clinic-visiting data to the corresponding network and calculate the minimum spanning tree. Finally, a logistic regression model trained by data consisting of MST-DNM scores was applied to detect the tipping point of influenza for each year.

As shown in Figures S1 and S2 of Supplementary Information (see Supplemental File), the critical state of the influenza outbreak was smoothly detected by our method MST-DNM in Hokkaido between 2011 and 2015 and in Osaka between 2012 and 2017, respectively. In other words, the MST-DNM method is quite general and robust irrelevant to the scale of the city network. The dynamic evolutions of the minimum spanning tree of Hokkaido city network and Osaka city network are shown in Figures S3 and S4, respectively.

### 3.3. The Key Role of the Minimum Spanning Tree

**Figure 5:**
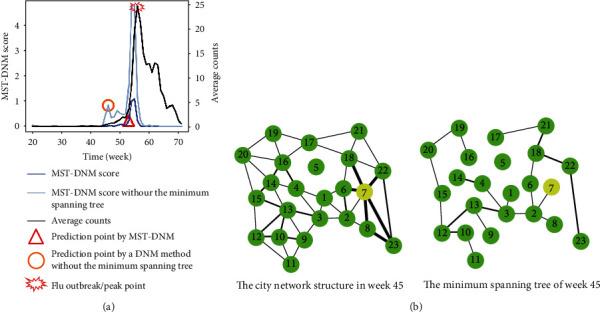
The comparison result of the MST-DNM method on the presence or absence of the minimum spanning tree in 2010. (a) The early-warning signal of a DNM method without the minimum spanning tree is far away from the real influenza outbreak point; however, the MST-method's is measurable. (b) The minimum spanning tree avoids abnormal correlations around node 7 in week 45, through which the MST-DNM method is more accurate.

In order to demonstrate the key role of the minimum spanning tree in our approach, we compared the effect of the MST-DNM method on the presence or absence of the minimum spanning tree in 2010, which is presented in Figure 5(a). It can be seen that the early-warning signal detected by a DNM method without the minimum spanning tree is far away from the influenza outbreak point but another signal appears in an appropriate time point.

An undirected and edge-weighted minimum spanning tree is the smallest tree model that minimizes the sum of the weights of all connected edges in the original network. It is able to reflect the overall changes of the network structure and could avoid the impact caused by local abnormal correlations around node 7 in week 45, which indicates that the minimum spanning tree plays a key role in the prediction process of outbreak points.

### 3.4. Performance Comparison with Other Methods

**Figure 6:**
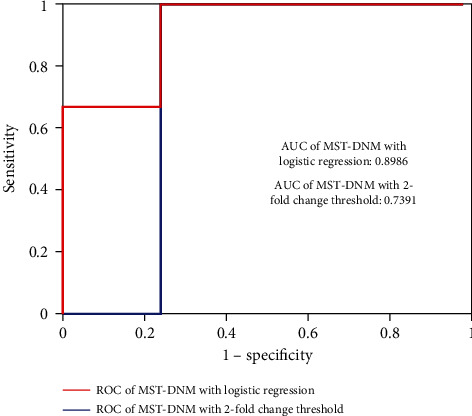
The performance of the MST-DNM method in different critical status determination strategies, that is, logistic regression and 2-fold change threshold. It can be seen that the MST-DNM method based on logistic regression is better than that based on 2-fold change threshold. The AUC of MST-DNM with logistic regression is 0.8986 while that of MST-DNM with 2-fold change threshold is 0.7391.

In the previous work, we developed a groundbreaking network-based approach for predicting influenza outbreaks, the so-called landscape dynamic network marker, which used empirical fold-change threshold to recognize the significant changes in DNM score to get the early-warning signal. We compared the performance of the proposed method MST-DNM with different tipping point determination strategies, that is, threshold determined from logistic regression and empirical threshold, which is presented in Figure 6. It is clear that the performance of the MST-DNM method based on logistic regression is better than that on the fold-change threshold. Actually, the logistic regression has natural advantages relative to the traditional early-warning signal determination methods. The logistic regression model is a more general and more robust method only with some appropriate training measures.

## 4. Conclusions

Japan suffered a serious influenza outbreak at the beginning of year 2019. According to the reports of about 5000 designated medical institutions across Japan, there was an average of 57.09 influenza patients per institution in the week from January 21st to 27th, which hit a new historical high since the first statistics in 1999. The influenza epidemic causes school suspension and the absence of a large number of workers, which would further result in a decline in social productivity and affect the economic development. It is estimated that the direct economic losses caused by the 2009 influenza pandemic to countries are about 0.5% to 1.5% of gross domestic product (GDP) [24]. However, the actual losses may be higher, due to the underestimate for the indirect economic losses caused by other infection prevention and control measures, such as the decline of tourism. Therefore, in order to better prevent the outbreak of influenza, it is quite essential to establish a real-time monitoring system only based on available and robust data, such as the number of clinic visits issued by the relevant health department.

Based on the DNM theory, which was applied to detect the tipping point or analysis critical transition of complex diseases on related genomic data in our previous works, combined with minimum spanning tree and logistic regression, a novel computable method called MST-DNM was developed to identify the early-warning signal of influenza outbreak in Tokyo, Osaka, and Hokkaido of Japan. In our MST-DNM method, we first extract the crucial characteristics of the preoutbreak state of influenza using DNM and minimum spanning tree from high-dimensional and longitudinal clinic-visiting counts. Then, the logistic regression trained by leave-one-out cross-validation is applied to identify the preoutbreak state and issue an early-warning signal based on these crucial characteristics. As shown in Figures 3 and 4, the MST-DNM method could timely detect the early-warning signal of influenza outbreak, which makes it quite possible to construct a real-time and effective influenza surveillance system. Nevertheless, there are still a few ways to improve the performance of our algorithm, such as using other robust but hardly obtainable data like population movement between wards and flu epidemic report to calculate the Pearson correlation coefficient and standard deviation, which is one of our future topics.

## Figures and Tables

**Algorithm 1:**
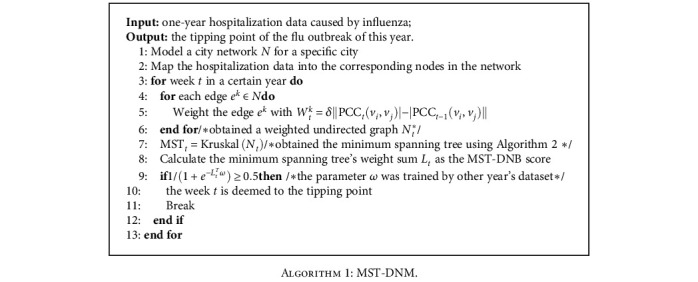
MST-DNM.

**Algorithm 2:**
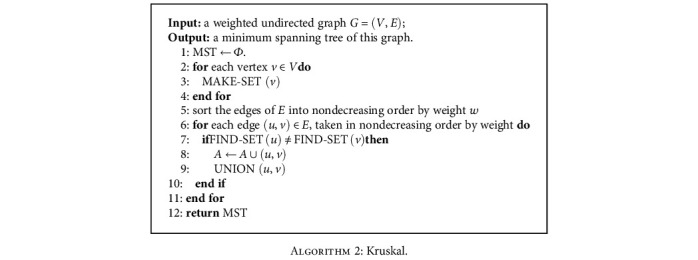
Kruskal.

## Data Availability

The historical raw data is available from Tokyo Metropolitan Infectious Disease Surveillance Center (link: https://survey.tokyo-eiken.go.jp/epidinfo/weeklyhc.do), Hokkaido Infectious Disease (link: http://www.iph.pref.hokkaido.jp/kansen/501/data.html), and Osaka Infectious Disease (link: http://www.iph.pref.osaka.jp/infection/2-old.html), respectively.

## Supplementary Materials

Supplementary Materials Figure S1: the predictions of annual influenza outbreak in Hokkaido city between 2011 and 2015. Figure S2: the predictions of annual influenza outbreak in Osaka city between 2012 and 2017. Figure S3: the dynamic evolution of the minimum spanning tree of the city network in Hokkaido during years 2014-2015. Figure S4: the dynamic evolution of the minimum spanning tree of the city network in Osaka during years 2017-2018.
