# Supplementary material for: Real-Time Forecast of Influenza Outbreak Using Dynamic Network Marker Based on Minimum Spanning Tree
Source: Biomed Res Int. 2020 Oct 1;2020:7351398. doi: 10.1155/2020/7351398 (PMC7547339; doi:10.1155/2020/7351398)
Supplement: Supplementary Materials — Figure S1: the predictions of annual influenza outbreak in Hokkaido city between 2011 and 2015. Figure S2: the predictions of annual influenza outbreak in Osaka city between 2012 and 2017. Figure S3: the dynamic evolution of the minimum spanning tree of the city network in Hokkaido during years 2014-2015. Figure S4: the dynamic evolution of the minimum spanning tree of the city network in Osaka during years 2017-2018. [file 7351398.f1.pdf]

Supplementary Information of Real-time forecast of influenza outbreak using dynamic network marker based on minimum spanning tree

Kun Yang, Jialiu Xie, Rong Xie, Yucong Pan, Rui Liu, Pei Chen

Supplementary Figures

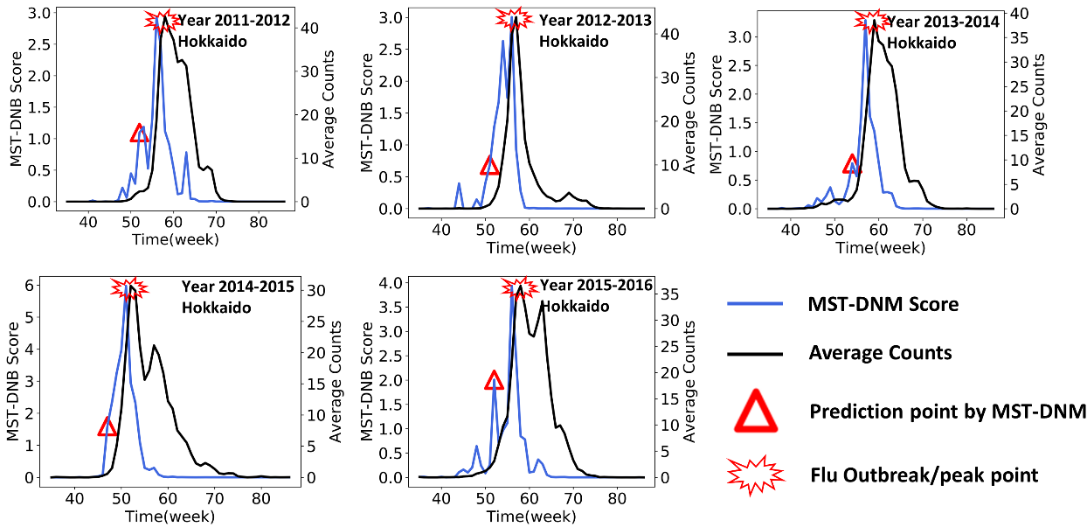

Figure S1: The predictions of annual influenza outbreak in Hokkaido city between 2011 and 2015. For each year, our MST-DNM method timely issues the early-warning signal of influenza outbreak only based on the clinic-visiting information. For each figure, the x-axis represents the time evolution from the 30th week to 85nd week (roughly a seasonal-outbreak period), the y-axis represents the MST-DNM score and average number of clinic visits, respectively. The red hollow triangle represents the early-warning signal detected by the MST-DNM method, and the explosion symbol is the actual outbreak point of influenza, i.e., the peak of the clinic-visiting number.

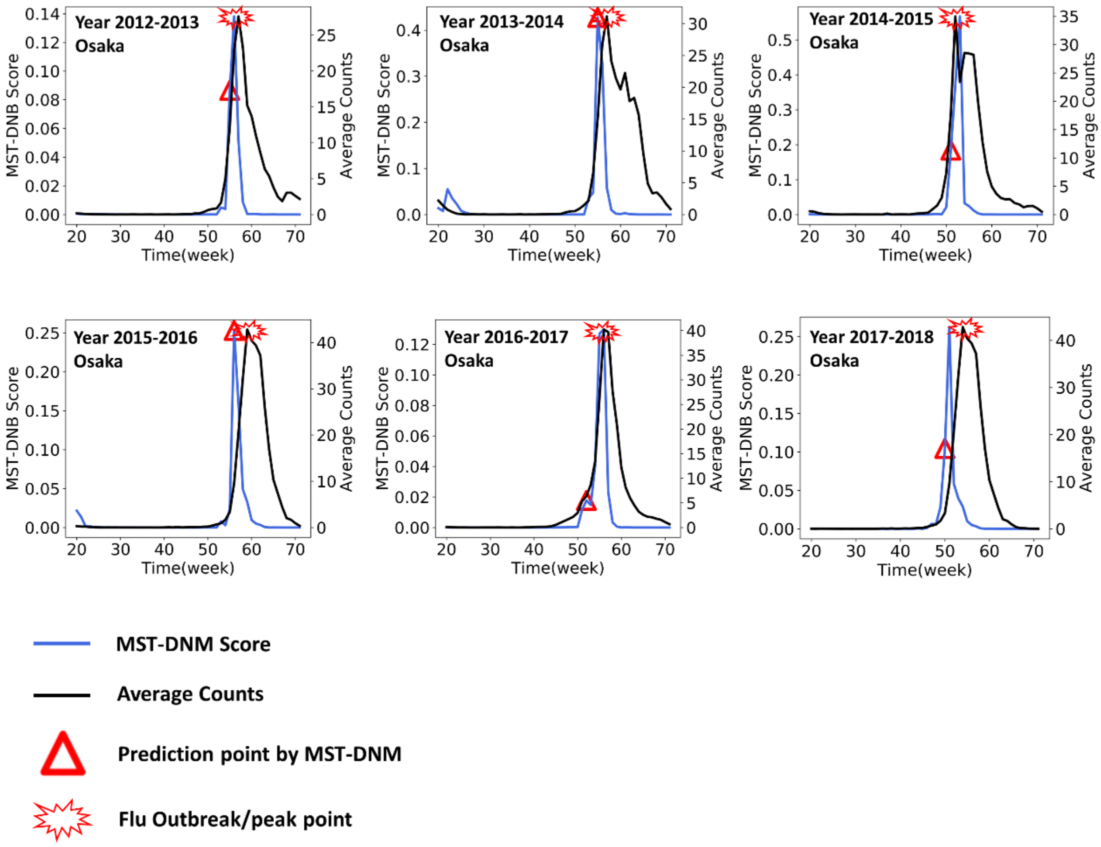

Figure S2: The predictions of annual influenza outbreak in Osaka city between 2012 and 2017. For each year, our MST-DNM method timely issues the early-warning signal of influenza outbreak only based on the clinic-visiting information. For each figure, the x-axis represents the time evolution from the 20th week to 72nd week (roughly a seasonal-outbreak period), the y-axis represents the MST-DNM score and average number of clinic visits, respectively. The red hollow triangle represents the early-warning signal detected by the MST-DNM method, and the explosion symbol is the actual outbreak point of influenza, i.e., the peak of the clinic-visiting number.

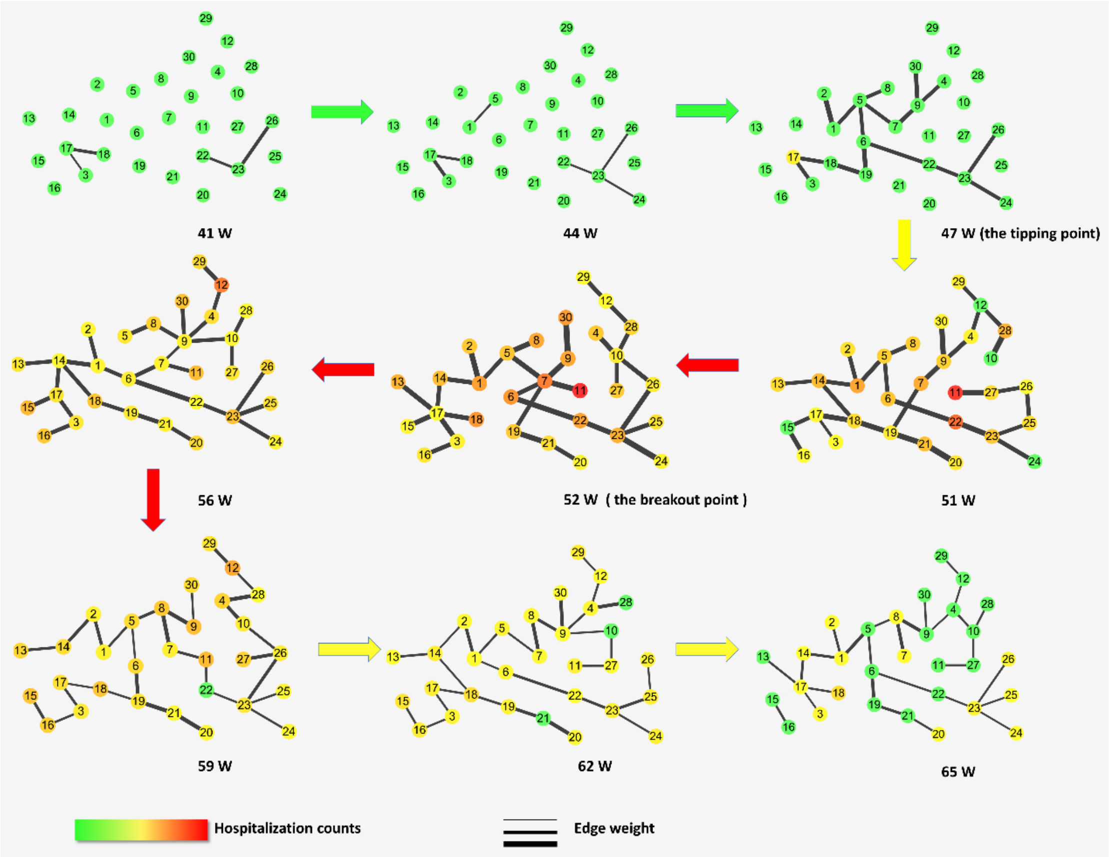

Figure S3: The dynamic evolution of the minimum spanning tree of the city network in Hokkaido during years 2014-2015. The nodes are colored by the average number of clinic visits of the corresponding district, and the thickness of the edges represents the correlations between corresponding nodes. It's clear that the edges become thicker before the nodes turn red in week 47, which indicates that the early warning signals from our method appears before the flu outbreak.

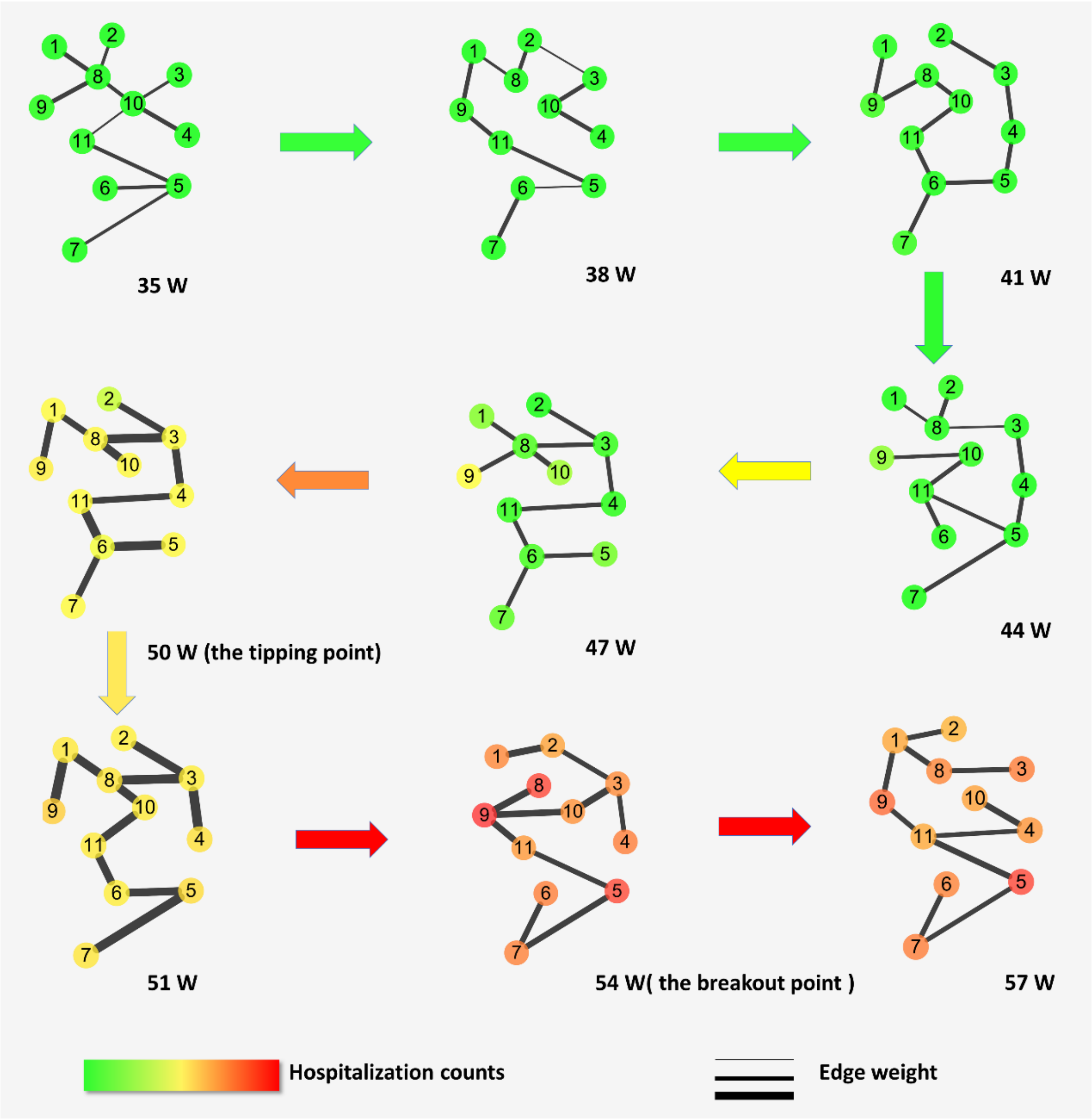

Figure S4: The dynamic evolution of the minimum spanning tree of the city network in Osaka during years 2017-2018. The nodes are colored by the average number of clinic visits of the corresponding district, and the thickness of the edges represents the correlations between corresponding nodes. It's clear that the edges become thicker before the nodes turn red in week 50, which indicates that the early warning signals from our method appears before the flu outbreak.
